# Investigation of the Foam Development Stages by Non-Destructive Testing Technology Using the Freeze Foaming Process

**DOI:** 10.3390/ma11122478

**Published:** 2018-12-06

**Authors:** Johanna Maier, Thomas Behnisch, Vinzenz Geske, Matthias Ahlhelm, David Werner, Tassilo Moritz, Alexander Michaelis, Maik Gude

**Affiliations:** 1 Institute of Lightweight Engineering and Polymer Technology (ILK), Technische Universität Dresden, Holbeinstr. 3, 01307 Dresden, Germany; thomas.behnisch@tu-dresden.de (T.B.); vinzenz.geske@tu-dresden.de (V.G.); maik.gude@tu-dresden.de (M.G.); 2 Fraunhofer Institute for Ceramic Technologies and Systems (IKTS), Winterbergstraße 28, 01277 Dresden, Germany; matthias.ahlhelm@ikts.fraunhofer.de (M.A.); david.werner@ikts.fraunhofer.de (D.W.); tassilo.moritz@ikts.fraunhofer.de (T.M.); alexander.michaelis@ikts.fraunhofer.de (A.M.)

**Keywords:** Freeze Foaming, in situ computed tomography, non-destructive testing, bioceramics

## Abstract

With a novel Freeze Foaming method, it is possible to manufacture porous cellular components whose structure and composition also enables them for application as artificial bones, among others. To tune the foam properties to our needs, we have to understand the principles of the foaming process and how the relevant process parameters and the foam’s structure are linked. Using in situ analysis methods, like X-ray microcomputed tomography (µCT), the foam structure and its development can be observed and correlated to its properties. For this purpose, a device was designed at the Institute of Lightweight Engineering and Polymer Technology (ILK). Due to varying suspension temperature and the rate of pressure decrease it was possible to analyze the foam’s developmental stages for the first time. After successfully identifying the mechanism of foam creation and cell structure formation, process routes for tailored foams can be developed in future.

## 1. Introduction

The two conventional processes for manufacturing ceramic cellular foam structures are the replica, as well as the space holder method [1,2]. These methods use organic scaffolds, which have to be burnt out. A novel manufacturing route for ceramic foam structures, called Freeze Foaming, that avoids the use of organic additives, has been developed by the Fraunhofer Institute for Ceramic Technologies and Systems (IKTS) [3,4]. The cell structure of a sample manufactured using Freeze Foaming is defined by a pressure-induced and pressure-controlled foaming process, followed by subsequent freeze drying, of a ceramic suspension in a vacuum. There are two different foaming agents—as ambient pressure drops, a reduced boiling point leads to the evaporation of water out of the aqueous suspension. The other one is air that is introduced during the manufacturing of the suspension. While the pressure is reduced (and the foam expands), the suspension’s temperature follows the line of equilibrium in the phase diagram of water to the triple point. Since the pressure is reduced further, the temperature falls beneath the equilibrium temperature in the triple point of our suspension, which causes our created structure to be instantly frozen, and dries via sublimation. This freezing step can result in cryogenic structures similar to typical freeze cast structures [5,6] and accounts for the microporosity of foamed structures. Possible applications of foams and porous parts made using Freeze Foaming encompass a wide spectrum, including biomedical uses, like artificial bones [7,8,9] as well as carrier materials for catalytic converters [10], biosensors and drugs, or thermal [11] and acoustic insulators [12]. Freeze Foaming enables the processing of biocompatible materials while offering the unique possibility of creating foams exhibiting a multimodal pore-size distribution and interconnectivity. These factors offer good conditions for the cultivation of organic cells. Previous work [3,4,7,13] has shown a particular suitability for an application in artificial bones due to their special structural properties. 

As the foaming is influenced by a complex interaction of several process and material parameters, further research into the foam formation during Freeze Foaming is needed. A reproducible manufacturing of tailored foams with a specified structure is not possible as of now. To adjust the properties according to applications, and develop process and quality guidelines, it is important to further examine the influence of relevant process parameters on the foam’s structure. With the presented work, the authors aim at finding a solution to a tunable shaping method, which enables the manufacturing of highly controllable pore structures to be used, e.g., as bone-mimicking scaffolds for an ever-older population [14].

To that end, an in situ µCT extension for an existing scanner was developed, which allows the analysis of different steps of the very foaming process during the manufacturing. Conventionally, examining changes in a sample using X-ray computed tomography is done by scanning before and after a change in state or structure [15,16]. The conventional method does not enable observation of the foam development of Freeze Foams. In material research focusing on damage and degradation analysis, significant improvements were made in the last years, due to progressive in situ techniques [15,17,18]. 

In the investigations from [19], the sintering process of ceramics could be analyzed with the use of CT. It was sufficient to analyze the process every 30 min to get a statement about the sintering theories. Further investigations in the field of in situ analyses are described in [20]. In so-called in situ X-ray nanotomography systems, pixel sizes of 100 nm are achieved in 20 s, with the use of a focus size of 50 nm. The sintering stages of metals and ceramics were also analyzed. In the investigations presented here for the analysis of the formation process of Freeze Foams, such a resolution is not necessary. In the investigations from [21], the hydration of gypsum plaster setting was investigated with in situ X-ray tomography. The scan duration was 200 s. However, the entire structure formation of the Freeze Foaming process (i.e., foaming and freezing) takes only about 60 s. Therefore, a novel CT setup had to be developed, firstly, in order to visualize the foaming process per se and, secondly, to introduce measures making tomographic image acquisition possible. The process had to be designed in such a way that it could be stopped at certain process steps and fixed for the CT imaging. This entire experimental setup—a new controllable laboratory freeze dryer in a computer tomography scanner—is one of the fundamental novelties of this work. In the first phase, a process-optimized testing device was developed [22]. It is suitable for 2-dimensional examinations using X-ray radiography (for real-time observation of the foaming progress), as well as three-dimensional scans to evaluate structural phenomena. Using the now-reproducible manufacturing of a model suspension [23], detailed results of in situ foam structure analysis are presented.

## 2. Materials and Methods 

The ceramic suspensions used in this work are composed of water and dispersion agent (Dolapix CE 64, Co. Zschimmer & Schwarz Mohsdorf GmbH & Co. KG, Burgstädt, Germany), added hydroxyapatite powder (Sigma-Aldrich now Merck KGaA, Darmstadt, Germany; BET = 70.01 m^2^/g, d_50_ = 2.64 µm), binder (polyvinyl alcohol), and rheological modifier (Tafigel PUR40, Co. Münzing Chemie GmbH, Heilbronn, Germany) [23]. The choice of suspension and composition was derived from preliminary tests on the basis of different suspensions which, after Freeze Foaming, resulted in reproducible foam structures [23]. The detailed manufacturing process of the suspension is described in [24]. For the investigations of this contribution and with regard to its possible influence on the foaming process and structure formation, three ceramic suspensions with different temperatures were used (5, 23, and 40 °C).

**Figure 1:**
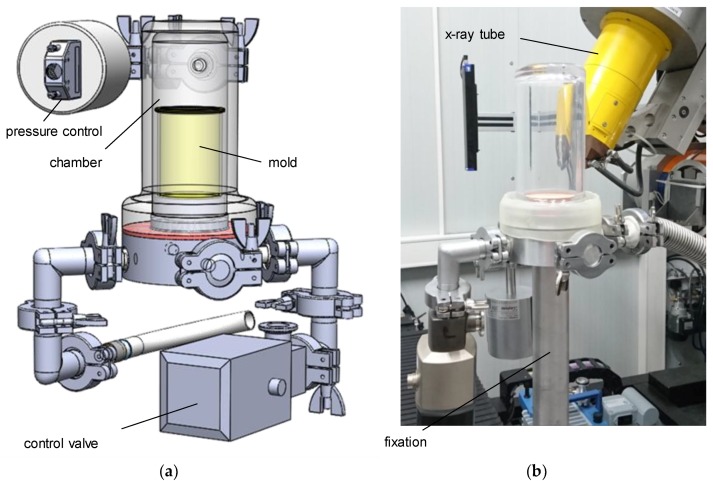
In situ µCT device: CAD model (**a**) and mounting situation (**b**).

An in situ device, to be used inside a v|tome|x L450 (General Electric, Cincinnati, OH, USA), was developed during the first phase of research [22]. It allows the material to be subjected to phenomenological analysis, and for detection and characterization of pores during the foaming process. To examine developmental steps during the formation of the foam’s structure, the device has to be leak-proof under vacuum (Figure 1). Using different foaming molds, the device can be used for either X-ray radiography (2D) or gaining spatial information (µCT, 3D) about the foam’s structure. The resolution was set to 22 µm/vx using an acceleration voltage of 100 kV, and a beam current of 300 µA. To fix the foaming suspension for the time of the CT scan (720 projections with 250 ms exposure time each, 3 min total measurement time), the foaming is stopped using a pressure control system with dedicated software, developed in-house, and an adjustable bypass. The pressure is kept at a constant level for the duration of the CT, in order to stabilize the structure. The vacuum chamber itself is rotationally symmetrical, and made of low-absorbing polymer to ensure optimal image quality. The choice of polymethylmethacrylate (PMMA) for the chamber prevented stabilizing the foam by means of externally freezing, as the material’s thermal conductivity is very low.

**Figure 2:**
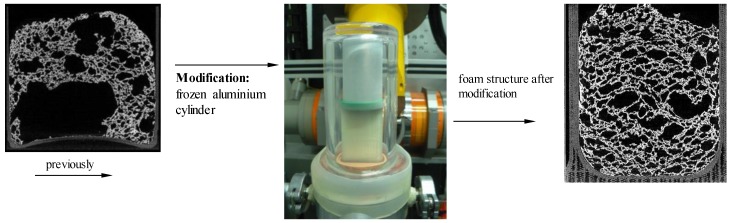
Improvements to the experiment.

In previous examinations [22], the pressure reduction rate’s influence on the foam structure—and especially the orientation of pores—has been shown. Due to the concave nature of the bottom of the mold, foaming led to a high number of samples exhibiting large pores near the bottom, which distorted the results of the foam analysis. Therefore, a new mold with a flat bottom was designed (Figure 2). Furthermore, water vapor emitted from the suspension decreased the pressure reduction rate in the lower pressure range of 25 mbar and below (near equilibrium of water vapor at 20 °C). The reduced pressure drop led to a higher amount of coalescence effects in the finished foam structure. To accelerate the pressure reduction, a cold trap has been used. For this purpose, a cylinder made from aluminum, with channels, was manufactured. It was cooled down with liquid nitrogen and placed on top of the mold (Figure 2, right picture). The water vapor condenses on its surface, which significantly reduces the time to fall below the triple point.

Besides the process analysis compression tests, in situ µCT scans under compressive load were also conducted on the conducted foams. In situ compressive scans were performed using a Finetec FCTS 160 IS (Garbsen, Germany) with an acceleration voltage of 50 kV and a beam current of 250 µA. The resolution was around 11 µm/vx, and the exposure time 625 ms. Mechanical testing of prepared cylindrical samples took place using an universal testing machine Zwick 1475 (Ulm, Germany). A preload of 2 N and a traverse speed of 2 mm/min were chosen.

## 3. Results

### 3.1. Radiographical Evaluation of the Freeze Foaming Process

**Figure 3:**
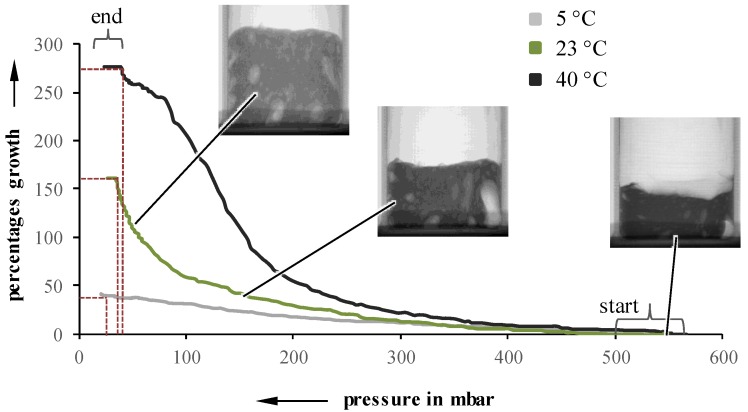
Percentage growth as a function of pressure for 5, 23, and 40 °C, at 10 mbar/s.

By acquiring two 2D pictures per second (500 ms exposure time) a real-time observation and analysis of the foaming process is possible. Due to the superposition of structural phenomena, the thickness of the sample was reduced to 5 mm for radiographically evaluations. To qualify the changes between the steps, manual tracking is conducted by overlaying each picture with a grid, and following the movement of distinct points in the sample. The resulting coordinates can be converted to changes in actual values for the size or height of the foam, and can be correlated with the pressure at the moment of picture acquisition. This method of evaluation was applied for three different sample temperatures and a pressure reduction rate of 50 and 10 mbar/s, respectively. As an example, the plotted results for 10 mbar/s are shown in Figure 3, as experiments at different pressure reduction rates behave similarly.

**Figure 4:**
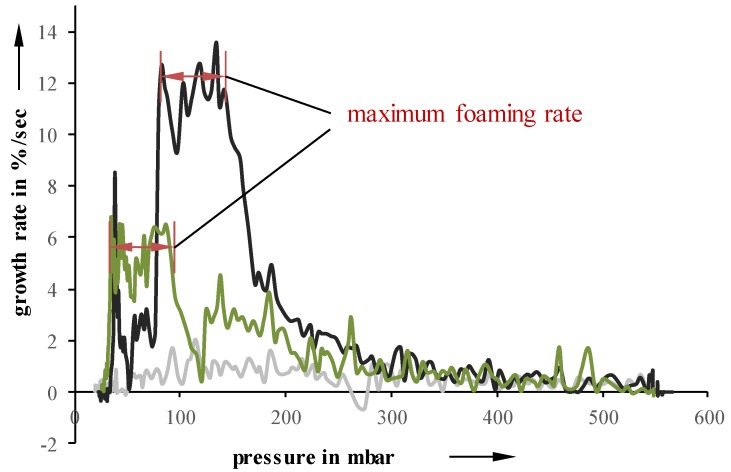
Foaming rate as a function of pressure for different temperatures at 10 mbar/s.

Independent of the initial suspension temperature, foaming starts between 450 and 550 mbar. However, the growth, as well as the pressure at which the foaming stops, are highly influenceable by the initial temperature. A lower temperature leads to an inhibited foaming, which results in a lower overall growth. Even though the foaming process itself continues up to lower pressures, the lower foaming rate cannot be compensated. For example, a suspension with a pressure reduction rate of 10 mbar/s and an initial temperature of 5 °C stops foaming at 15 mbar, while a 40 °C suspension already stops at 35 mbar. Suspensions undergoing 50 mbar/s exhibit a very similar behavior. Looking at foaming rates over pressure, the highest suspension temperature also results in the highest growth rates values at higher pressures (Figure 4). 

Samples with an initial temperature of 23 °C exhibit a lower maximum at lower pressures while 5 °C samples showed an almost constant foaming rate and, therefore, no identifiable maximum.

To evaluate and verify the findings from the radiographical evaluation, the results were compared to data obtained from the freeze dryer at IKTS (Table 1) [24]. Apart from 5 °C suspensions, their findings support the trend identified at the ILK. For suspensions with an initial temperature of 5 °C, the IKTS identified a foaming rate maximum at pressures between 40 and 60 mbar. However, the freeze dryer is equipped with an additional condenser, which is not available in the in situ device. It is not possible, so far, to achieve the foaming rate maximum and finish the pressure-induced foaming process.

**Figure 5:**
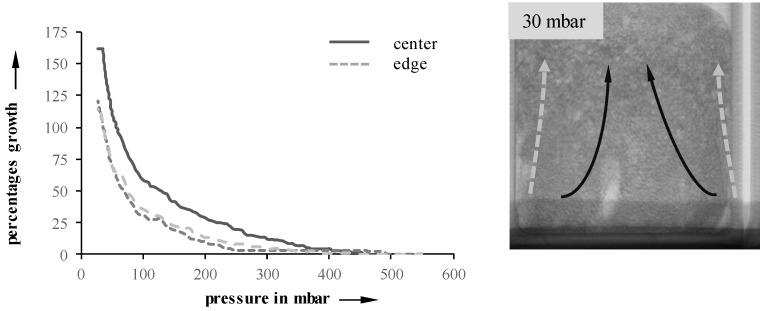
Edge effects on foaming at 23 °C (10 mbar/s).

Through analysis of radiographic images, edge effects on the foaming process can be verified. Figure 5 illustrates their impact on a 10 mbar/s sample at 23 °C. Both edges, as well as the center, were manually tracked using the method described earlier, and the local growth rates were determined. As expected, the edges exhibit a much slower growth when compared to the center. Possible reasons include wall friction, as well as a drying of the suspension. This behavior is especially observable in suspensions with an initial temperature of 40 °C, which also develops a compact layer on top of the suspension.

### 3.2. CT Evaluation of the Freeze Foaming Process

To examine the developmental stages of foaming, µCT and the improved testing device is used to create a virtual and reconstructed volume (VGStudio 2.0, Volume Graphics GmbH, Heidelberg, Germany) of the foam’s structure. During the CT measurements, the sample is rotated 360°, and after an angle of 0.25°, one image is taken. Those pictures can be reconstructed with the program “Phoenix datos”, which is generating a 3D model. This model can be imported into “VGStudioMax 3.0” (Volume Graphics GmbH). In this program, it is possible to perform various analyses, such as defect analysis or foam structure analysis. A region of interest (ROI) is selected, and a surface determination is performed automatically. A threshold value is determined to be able to separate material from background. The porosity can be determined from this data. 

In order to acquire sufficient CT data, given that Freeze Foaming is a fast process, the exposure time is reduced from 500 to 250 ms. In addition, the number of images for the holding steps is reduced from 1440 to 720. The following parameters were selected for the evaluation of the foam structure: Threshold—80%, Accuracy—Fast; Direction of analysis—Right; Analysis mode—Background; Features—Advanced cell properties.

**Figure 6:**
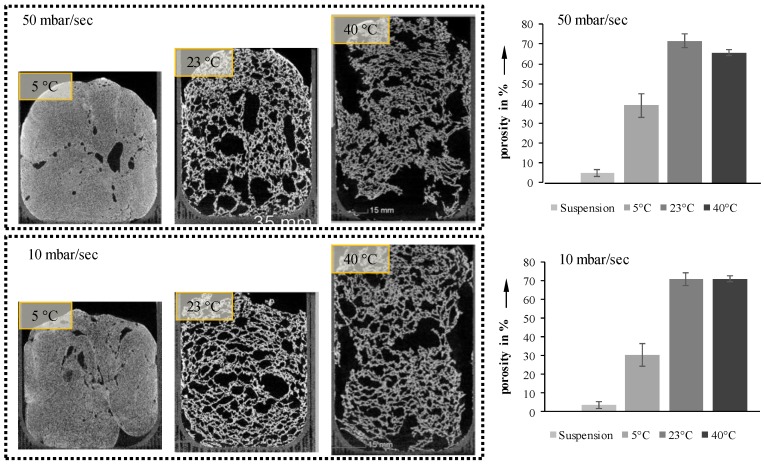
Cross-sections and porosities of completely foamed suspensions at two pressure reduction rates and three temperatures.

The first step was a complete foaming at two pressure reduction rates (10 mbar/s and 50 mbar/s). The results and their porosities are shown in Figure 6.

Increasing the pressure reduction rate from 10 to 50 mbar/s results in only a slight increase in porosity (Figure 6, right). However, the orientation of the pores seems to be significantly influenced by the pressure reduction rate. Suspensions foamed at 50 mbar/s exhibit vertically elongated pores, while those foamed at 10 mbar/s are oriented more horizontally. Due to the higher reduction rate inflicted on the process-induced air, the velocity of the inflating bubbles increases, thus forming vertical pores. In both cases, this is especially visible for 23 °C samples. Samples with an initial temperature of 40 °C show a decrease in porosity during foaming, due to their low viscosity, and the foam collapses before freezing by reaching the triple point.

### 3.3. Stages of the Foaming Process

**Figure 7:**
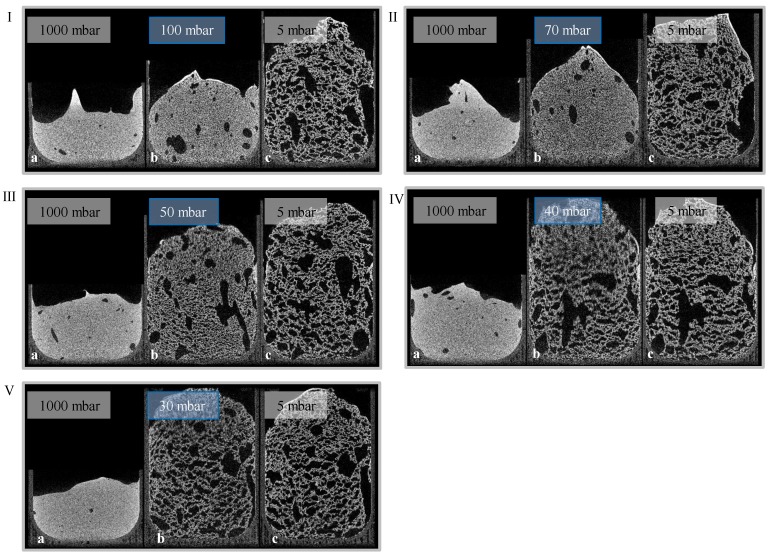
Holding stages (I: 100 mbar, II: 70 mbar, III: 50 mbar, IV: 40 mbar, V: 30 mbar) of a 23 °C tempered suspension with a pressure reduction rate of 10 mbar/s ((**a**) suspension; (**b**) holding stage; (**c**) foam).

**Figure 8:**
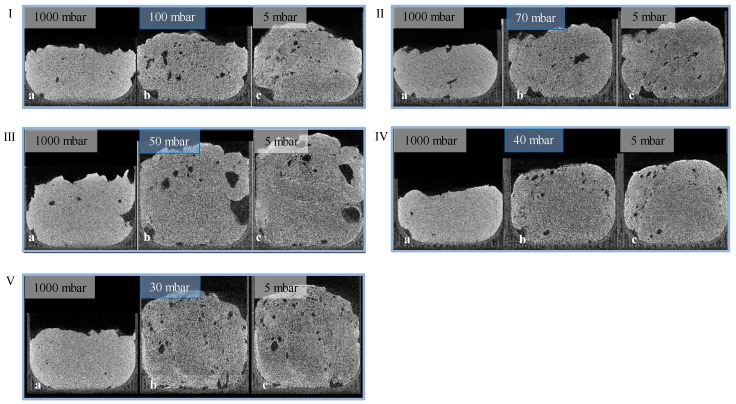
Holding stages (I: 100 mbar, II: 70 mbar, III: 50 mbar, IV: 40 mbar, V: 30 mbar) of a 5 °C tempered suspension with a pressure reduction rate of 10 mbar/s ((**a**) suspension; (**b**) holding stage; (**c**) foam).

Given the results of radiographic imaging, five holding stages were identified for analyzing the stages of the foaming progress (at 30, 40, 50, 70, and 100 mbar). The pressure reduction rate was adjusted to 10 mbar/s. Cross-sections of those scans are shown in Figure 7 (5 °C) and Figure 8 (23 °C). For each evaluation, three CT scans were executed to observe pores, and their development—the suspension (1000 mbar), the holding stage at its target pressure, and the final foam structure (5 mbar). Air bubbles that have been introduced into the mold during suspension filling have a large influence on the foam structure. They grow even larger during foaming, and develop significantly larger pores. Due to their high viscosity, this growth is inhibited in 5 °C tempered suspensions. Furthermore, the maximum foaming rate takes place at lower pressures (40–60 mbar) [24]. As a large amount of water evaporates, the pressure reduction rate drops, and the time to reach the target pressure of <5 mbar is too long. This process-induced growth inhibition results in a significantly lower porosity. In general, suspensions with an initial temperature of 5 °C exhibit a lower porosity after foaming, due to a higher viscosity and a lower amount of escaping water vapor. On the other hand, suspensions with an initial temperature of 40 °C exhibit a viscosity too low to be stable during the CT scan and, therefore, were not monitored.

**Figure 9:**
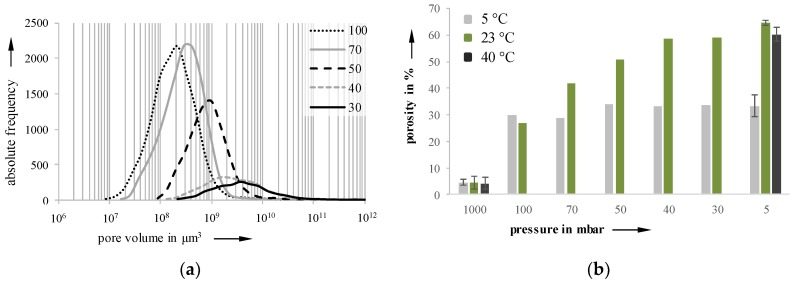
Pore size distribution of a 23 °C sample (**a**) and porosity (**b**) at 5, 23, and 40 °C samples of different holding stages; pressure reduction rate: 10 mbar/s.

Using the software VGStudioMax, which allows access to volume-based data, the pore size distributions were determined for each holding stage. An exemplary distribution for a 23 °C sample is depictured in Figure 9a. The growth starts slowly, accelerates to a maximum, and then slows down again. The lowest variance in pore size distribution can be found at 50 mbar on a 23 °C sample, with a relative curve width (b = d_90_/d_10_) of 36.2 (b_100mbar_ = 53.1, b_70mbar_ = 36.2, b_40mbar_ = 153.5, b_30mbar_ = 160.9). When the pressure drops below 50 mbar, the pore size distribution becomes flatter and wider, indicating a ripening process. Figure 9b shows the increasing porosity as a function of the decreasing pressure for the three investigated temperatures. Due to their high viscosity, 40 °C foamed samples could not be investigated with regard to holding stages and foam formation, because the foam structures collapsed during the investigation.

### 3.4. Mechanical Properties

**Figure 10:**
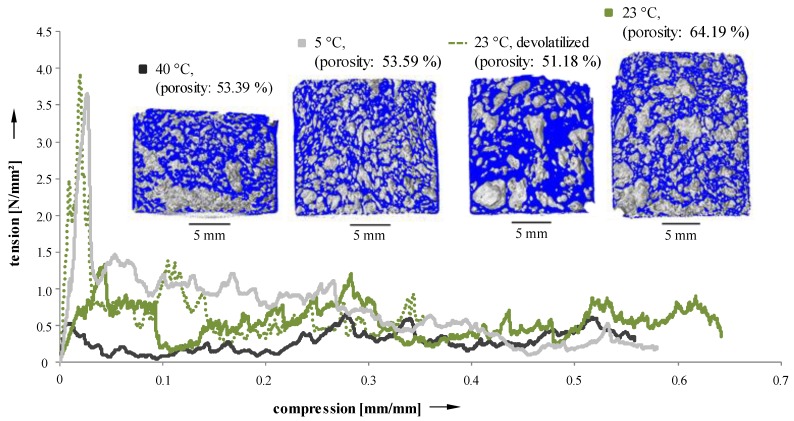
Compression tests on sintered samples (40 °C suspension; 5 °C suspension; devolatilized 23 °C suspension; 23 °C suspension).

Due to the dependence of porosity and pore size distribution of Freeze Foamed samples on process parameters, mechanical properties should vary as well. To evaluate their behavior under load, cylindrical sintered samples were manufactured and subjected to standardized compression tests. Recorded tension–compression curves are depicted in Figure 10. 

The fracture behavior clearly exhibits a dependency on suspension temperature (5, 23, and 40 °C) and pretreatment (devolatilized and not devolatilized suspensions during the manufacturing process). Samples with a narrow pore size distribution [24] (5 °C and 23 °C devolatilized [23]) possess a pronounced maximum of force. On the other hand, specimens with a less uniform distribution of pores [24] (40 °C and 23 °C not devolatilized) show a more constant force level, and only reach about half the maximum force when compared to more homogeneous samples. Mechanical properties of the samples manufactured using Freeze Foaming are strongly influenced by microporosities inside the struts [23]. However, as the resolution of the CT scans were insufficient to examine their structure, they could not be taken into account here.

**Figure 11:**
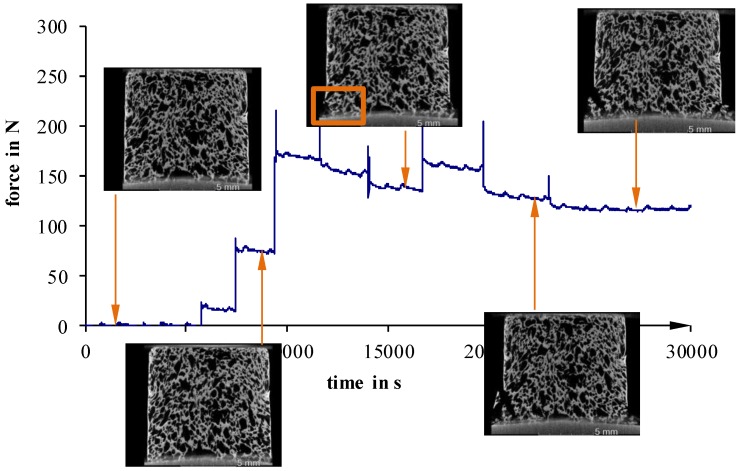
Recorded force–travel curves of compression tests conducted on an in situ µCT; 5 °C.

**Figure 12:**
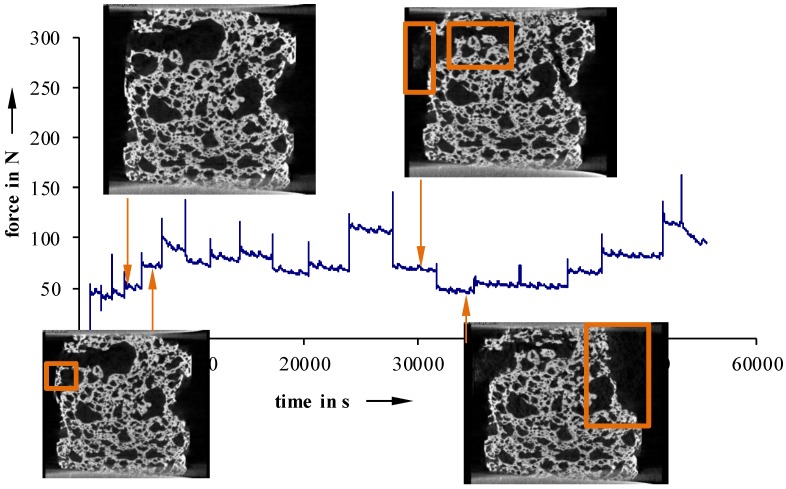
Recorded force-travel curves of compression tests conducted on an in situ µCT; 23 °C not devolatilized.

**Figure 13:**
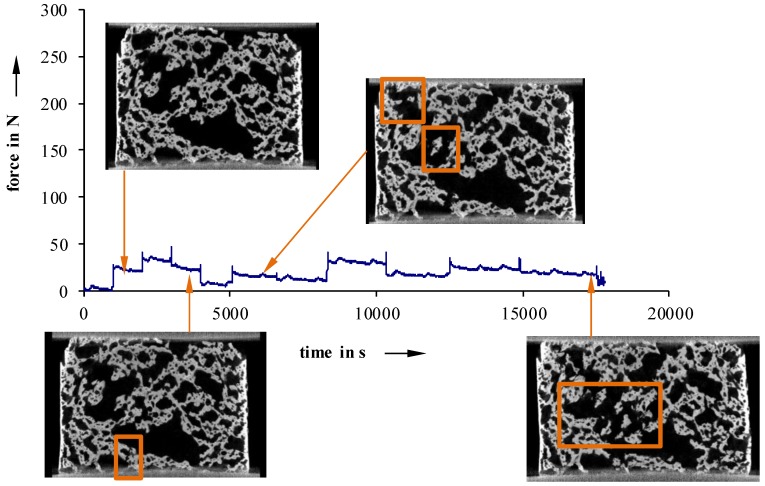
Recorded force-travel curves of compression tests conducted on an in situ µCT; 40 °C.

Furthermore, in situ compression tests, at selected load levels, were conducted to examine failure phenomenology (Figure 11, Figure 12 and Figure 13).

For more inhomogeneous samples, material failure starts to occur between 25 N (40 °C) and 50 N (23 °C) (Figure 12 and Figure 13). The density of fractures constantly rises, with increasing deformation, until a partial structure failure develops at relatively low load levels of 50 N (40 °C) and 110 N (23 °C not devolatilized). On the other hand, more homogeneous foam structures show a maximum load up to 200 N, even after the first signs of material failure (Figure 11). Detailed examinations show that especially cracks on the surface lead to material failure. This is a sign of an uneven sample surface, and results in a non-uniform load.

## 4. Conclusions

Biocompatible new materials will become increasingly important in the future. Ceramic structures based on Freeze Foaming allow ecological manufacturing without a need for organic scaffolds. Tailoring these ceramic foams to specific applications, a defined and reproducible adjustment of their structure and mechanical properties is necessary. However, the formation of the foam structure during Freeze Foaming is not yet fully understood. Their manufacturing is influenced by a complex interaction of different steps and material’s properties within the process.

Using a novel in situ µCT device, it was possible to examine the foaming process and the stages of the foaming process. Due to an integrated pressure control system, the foaming could be stopped at any applied pressure. 

Radiographic imaging gathered information about the beginning, maximum, and end of foaming, depending on the temperature of the suspension. Independent of the temperature, foaming starts between 450 and 550 mbar. An earlier end of foaming was detected when increasing the suspension temperature to 40 °C, due to a higher water vapor partial pressure and a lower viscosity. Suspensions with an initial temperature of 5 °C did not exhibit a foaming maximum in our device, due to their high viscosity.

To observe the pore formation during Freeze Foaming, µCT scans were performed using the new µCT device. Virtual volumes of Freeze Foam scaffolds were created and analyzed. Foaming was executed with varying pressure reduction rates. While the porosity changed only slightly with varying pressure reduction rates, the pores were oriented differently. During foaming, 40 °C tempered suspensions collapsed before reaching the triple point, due to their low viscosity. On the other hand, the growth of 5 °C suspensions were inhibited by their high viscosity. As a result of radiographic examinations, five pressure values were identified as holding stages of interest. Those stages revealed a large influence of air bubbles introduced during mold filling on the final foam structure. Independent of the initial temperature of the suspension, there is a continuous rise in porosity during the foaming process, in general, while the variance of pore size increases. Furthermore, the results of compression testing of sintered samples show a distinct force maximum for 5 °C and 23 °C tempered and devolatilized Freeze Foams. On the other hand, samples with a less homogeneous structure (40 °C and 23 °C not devolatilized) exhibit a force plateau and a maximum force about half that of samples.

Approaches for the defined production of Freeze Foams have been achieved. However, the complexity of the Freeze Foaming process requires more experiments and evaluation, in order to truly control the pore structure and, thus, make them more suitable for larger industries and applications.

## Figures and Tables

**Table 1:** Comparison of the pressures of beginning, end, and maximum of foaming at temperatures of 5, 23, and 40 °C by in situ µCTs (ILK) and by freeze dryer (IKTS) [24].

Suspension’s Temperature (°C)	Pressure at Beginning of Foaming (mbar)		Pressure at Maximum Foaming Rate (mbar)		Pressure at End of Foaming (mbar)	
	Freeze Dryer	In Situ µCT	Freeze Dryer	In Situ µCT	Freeze Dryer	In Situ µCT
5	500	450–550	40–60	n.d.	10	15
20	400	450–550	80	50–90	20	25
40	400	450–550	80–100	90–150	60	35
